# Supplementary material for: Membrane Association and Topology of Citrus Leprosis Virus C2 Movement and Capsid Proteins
Source: Microorganisms. 2021 Feb 17;9(2):418. doi: 10.3390/microorganisms9020418 (PMC7922530; doi:10.3390/microorganisms9020418)
Supplement: Supplementary file 1 [file microorganisms-09-00418-s001.pdf]

Figure

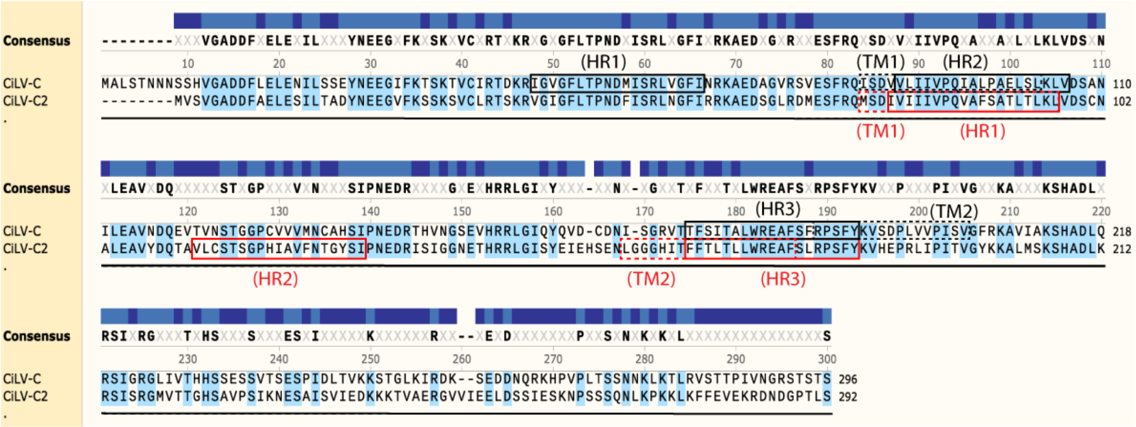

Figure S1. Amino acids sequence alignment of CiLV-C and CiLV-C2 MPs. Residues common at the two sequences are shown in blue (amino acids letters) and light blue (bar), whereas dissimilar residues are shown in white amino acids letters or dark blue (bar). The numbers on the right indicate the total size of the MP proteins. Virus acronyms are shown to the left of the image. The amino acids consensus is shown. The hydrophobic residues (HR) predicted using computer tool MPEx for CiLV-C MP (black box) and CiLV-C2 MP (red box) are presented. Transmembrane residues (TM) for CiLV-C MP and CiLV-C2 MP are indicated by black and red dotted box, respectively. The alignment was performed using the software SnapGene version 4.3.10.

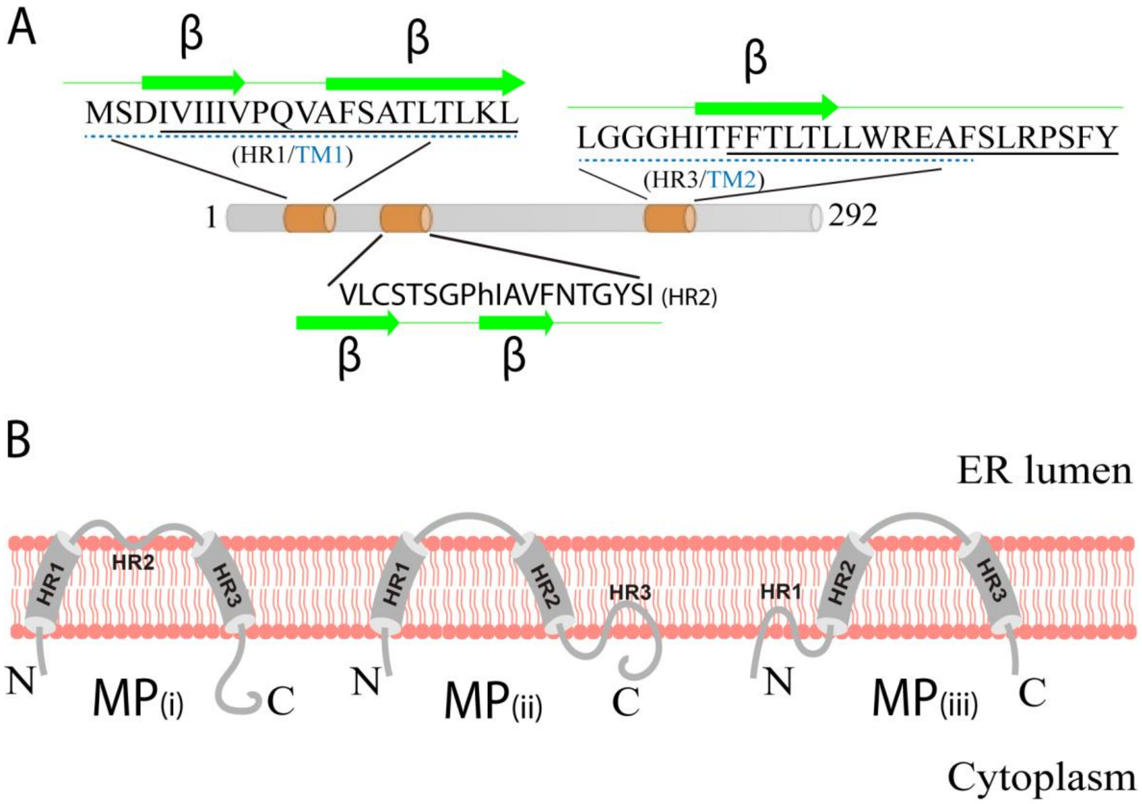

Figure S2. Topologic models of the association between CiLV-C2 MP and cell membranes. **A)** A schematic representation of the CiLV-C2 MP highlighting the HRs (residues underlined in black), TM segments (residues dashed in blue) and secondary structures (green arrows represent beta strands) predicted. The annotated secondary structural features were predicted using the PSIPRED algorithm. **B)** MP(i), represents the model that suggest HR1 and HR3 regions as TM segments and HR2 associated peripherally to the membrane. MP(ii), represents the model that suggests HR1 and HR2 regions as TM segments and HR3 associated peripherally to the membrane. MP(iii), represents the model that suggests HR2 and HR3 regions as TM segments and HR1 associated peripherally to the membrane.

Tables

Table S1. *In silico* analysis of the presence of transmembrane (TM) or hydrophobic regions (HR) of Lep, CiLV-C MP, CiLV-C2 MP and p29, TMV 30K MP, CSNV NSm and GFP amino acid sequences.

			TM positions			Hydrophobic region positions			
Protein	Algorithm	N °of TM segments	TM1/ΔG	TM2/ΔG	TM3/ΔG	HR1/ΔG	HR2/ΔG	HR3/ΔG	HR4/ΔG
Lep (integral)	MPEx	2	5-25/-3,1	80-102/-1,2		6-24/15,8	80-109/7,7	120-138/0,1	276-306/1,8
	△G prediction	2	5-25/-3,1	80-102/-1,2					
	HMMTOP	2	8-25	81-98					
	OCTOPUS	2							
	SOUSI	3	6-27	81-103	115-137				
	TMHMM	2	5-24	81-103					
	TMpred	3[a]/2[b]	6-25	84-103	284-305				
CiLV-C MP (Integral)	MPEx	0				48-66/0,2	88-106/2,0	173-191/1,7	
	△G prediction	0							

	HMMTOP	1	189-205				
	OCTOPUS	0					
	SOUSI	0					
	TMHMM	0					
	TMpred	1[a]/0[b]	84-103				
CiLV-C2 MP	MPEx	2	76-98/-1,7	160-178/-2,0	79-97/5,6	113-131/1,0	167-185/5,3
	△G prediction	2	76-98/-1,7	160-178/-2,0			
	HMMTOP	0					
	OCTOPUS	0					
	SOUSI	0					
	TMHMM	0					
	TMpred	2	79-98	159-180			
TMV 30K MP (peripheral)	MPEx	1	61-80/-2,4		148-166/2,0		
	△G prediction	1	61-80/-2,4				
	HMMTOP	1	149-166				
	OCTOPUS	0					

	SOUSI	0				
	TMHMM	0				
	TMpred	2[a]/1[b]	61-80	150-166		
CSNV NSm	MPEx	0			127-145/5,5	174-192/4,3
	△G prediction	0				
	HMMTOP	0				
	OCTOPUS	0				
	SOUSI	0				
	TMHMM	0				
	TMpred	1[a]/0[b]	127-149			
CiLV-C2 p29	MPEx	0			3-21/0,2	178-196/1,0
	△G prediction	0				
	HMMTOP	0				
	OCTOPUS	0				
	SOUSI	0				
	TMHMM	0				

	TMpred	0	
GFP (soluble)	MPEx	0	54-72/6,3
	△G prediction	0	
	HMMTOP	0	
	OCTOPUS	0	
	SOUSI	0	
	TMHMM	0	
	TMpred	0	

[a] Strong preferred model of TM insertion

[b] Alternative model of TM insertion

Table S2. Protein pair combinations performed in the BiFC topology assay for CiLV-C2 p29 and MP.

MEMBRANE TOPOLOGY		N-YFPcyt	C-YFPcyt	N-YFPer	C-YFPer
Controls	N-YFPer		-		+
	N-YFPcyt		+		-
p29	p29-N-YFP		+		-
	p29-C-YFP	-		-	
	N-YFP-p29		+		-
	C-YFP-p29	-		-	
MP	MP-N-YFP		+		-
	MP-C-YFP	-		-	
	N-YFP-MP		+		-
	C-YFP-MP	-		-	

The symbols - and + correspond to the absence or presence of fluorescence signals, respectively. N-YFP and C-YFP correspond to the N- and C-terminus splits of the YFP protein, respectively.
